# Predicting cervical screening and HPV vaccination attendance of Roma women in Hungary: community nurse contribution is key

**DOI:** 10.1186/s12912-022-00813-5

**Published:** 2022-01-30

**Authors:** Annamária Pakai, Réka Mihály-Vajda, Zsuzsanna Kívés Horváthné, Krisztina Szabó Gabara, Eszter Basa Bogdánné, András Oláh, Miklós Zrínyi, Adrienn Siket Újváriné

**Affiliations:** 1 Szombathely University Campus, Faculty of Health, University of Pécs, Pécs, Hungary; 2 Faculty of Health Sciences, Institute of Health Insurance, University of Pécs, Pécs, Hungary; 3 Faculty of Pharmacy, University of Pécs, Pécs, Hungary; 4 Kaposvár Campus, University of Pécs, Pécs, Hungary; 5 Living Lab Based Smart Care Research Center, University of Pécs, Pécs, Hungary; 6 Faculty of Health, University of Pécs, Vörösmarty utca 4, 7621 Pécs, Hungary

**Keywords:** HPV, Screening, Vaccination, Roma, Women, Community nurse, Hungary

## Abstract

**Background:**

HPV screening/vaccination has been observed lower for ethic minorities. Understanding factors that predict and can improve attendance is therefore key. Hence, the aim was to identify causes, especially concerning the quality of the patient-provider relationship, that predict past HPV screening and vaccination turnout of Roma women in Hungary.

**Methods:**

Cross-sectional research design with self-developed, culturally sensitive questionnaire. A final, female Roma sample of 368 participants was randomly selected from census register. Community nurses contacted participants and distributed surveys. Surveys were mailed-in by participants. Bivariate logistic regression was used to predict former participation in HPV screening/vaccination.

**Results:**

Of the total sample, 17.4% of women attended at least one cervical screening and HPV vaccination in the past. Bad screening experience was positively associated with racially unfair behaviors of physicians. The odds of past attendance were 4.5 times greater if ‘no negative earlier experience’ occurred, 3.3 times likelier if community nurse performed screening/immunization and 1.6 times more probable if respondent felt ‘no shame’. Evaluating the screening/vaccination process painful, being only financially motivated and attendance involving a lot of travel decreased the odds of ‘no show’ by 50%, 40% and 41%, respectively.

**Conclusions:**

When considering the ratio of past cervical screening attendance, we conclude that our female Roma sample did not behave differently from the general population. We saw no evidence that racial mistreatment made any contribution to explaining cervical screening participation. Past positive screening experience and the quality of patient-provider relationship increased the odds of participation the most. Cancer of friends, pain, financial motivation and travel distance decreased odds of participation to a lesser extent. In order to improve future screening and immunization, community nurses should play more central and advanced role in the organization and implementation of such services specifically targeting Roma populations.

## Introduction

As reported by the World Health Organization, Human papillomavirus (HPV) is an established cause of cervical cancer and ranks # 4 globally of all cancers in women [1]. Screening and vaccination are therefore key to prevent the disease. However, screening and vaccination participation remain a challenge, especially for women of ethnic minorities [2]. While ethnic groups were reported to have become more active in HPV vaccination uptake, they are still lagging behind in follow-through [2]. We also recognize that cultural HPV awareness and knowledge have a strong influence on vaccination attitudes and outcomes [3, 4]. Besides ethnic affiliation and personal knowledge, religious orientations were also identified as either constraints or enablers of vaccination [5, 6]. Riza et al. [7] confirmed that socio-economic background and education were important facilitators of Pap smear testing and HPV vaccination. They argued that Roma ethnic origin was associated with misguided beliefs concerning cervical screening and the HPV vaccine. Jackson et al. [8] described cultural aversion against HPV vaccination within travelling Roma communities which may also be part of a larger public mistrust [9]. A number of studies stressed that concerns over vaccine safety was another major barrier of getting vaccinated [10, 12, 13]. Income (access to vaccine and healthcare) and actual logistics of obtaining the vaccine and seeing a healthcare professional also prevented seeking HPV vaccination [11]. Getrich et al. [14] reasoned that favorable healthcare provider attitudes were essential in the decision to get screened and immunized. However, when healthcare staff, particularly female healthcare professionals, were surveyed, studies reported lower than expected HPV vaccination alertness and patient immunization encouragement by healthcare staff [15–17]. To achieve better screening and vaccination coverage, the quality of the patient-provider relationship between members of the Roma community and nurses and doctors was considered critical for prevention and immunization success [18]. Why this relationship may be so essential was underscored by Ilisiu et al. [19] who observed non-ethic women choosing hospital services for HPV screening whereas the majority of female Roma sought attention from general practitioners. Finally, Vu et al. [20] underlined that research should investigate how clinicians’ views and actions towards minorities influence HPV testing and vaccination to help develop or improve culturally sensitive interventions.

In conclusion, while global HPV screening and vaccination rates have increased for ethnic groups, there are still barriers to fully utilizing preventive services. Obstacles have to do with cultural norms and expectations but are also related to socio-economic reasons. Academics emphasized conducting research that looks at the quality of interactions with health professionals and their impact on screening attendance. Therefore, this research aimed to explore causes that predict past HPV screening and vaccination attendance in Roma women in Hungary. Since cultural and healthcare provider contexts vary and may differ across countries, authors developed their own, culture specific instrument for the purposes of this research. Indicators revealed in the literature review informed and guided item development.

## Methods

The actual research design was based on a cross-sectional survey with the intent to identify factors associated with past participation in HPV screening/vaccination. Data was collected between July–September 2019. Potential participants received a mail contact and called researchers to indicate their willingness to join. Local community nurses were contacted and trained by the research team to identify participants using inclusion/exclusion criteria. The same nurses contacted participants, handed over the research instrument and supported subjects in responding to items if requested. To avoid respondent bias, nurses only explained the research instrument but did not partake in responding. Participants forwarded surveys postal paid to the research team in sealed and unidentifiable envelopes. Final data entered was anonym and researchers could not trace it back to the original respondent.

### Instrument

The research instrument was developed specifically for the purposes of the current research. Local community nurses with long personal experience working in Roma neighborhoods participated in the instrument development. Researchers also considered outcomes of prior local and international research published on the topic [21–24]. Statements pertaining to health screening attitudes of Roma women obtained from previous research were organized into a survey form. Sample items include “I attend cervical screening/HPV vaccination because I care about my health”, “I attend screening/vaccination because my GP recommended to do so” and “I don’t attend cervical screening/HPV vaccination because it is too expensive”. The full scale assessed personal behaviors concerning personal reasons, health reasons, influence of a healthcare personnel, influence of others, travel and time related difficulties, lack of incentives (financial), access to and availability of screening services and inconvenience of the screening procedure. Responses were recorded on a 5-point Likert scale (1 = absolutely disagree, 5 = absolutely agree). The final ‘scale’ included 17 items related to the dimensions above. Scale validity in this research was not tested and established. Authors refer to content validity since real-life community nurse expertise was used to develop relevant scale items. Reliability, as measured by Cronbach’s alfa, was 0.88 for the full scale. Score range is between 17–85, greater scores indicating more potential for attending cervical screening/HPV vaccination. In terms of the logistic regression, individual items and not the aggregate scale was utilized.

Besides demographics, we asked respondents about their financial status (below average, average and above average), their current health (1 = very bad, 5 = excellent) and if they had ever been to cervical screening and received HPV vaccine at the same time (0 = no, 1 = yes).

#### Sample

The study sample was randomly selected from Roma women living in seven cities of Zala county with a total population of 282 thousand [25]. Roma women were chosen as the target population because Roma represent the largest ethnic group in Hungary. Given that census data allows for identifing minorities, women with Roma origin and in the age range 25–65 years were selected and contacted by mail. A total of 500 potential participants were randomly selected from census who had initially been contacted. Acknowledging the difficulties referred to by Condon et al. [26] and for ease of sampling and data collection, women only with a permanent address were recruited. Out of the 500 initially approached, a final sample of 368 women returned a consent and responded to our instrument. Exclusion criteria were: 1) non-Roma origin,2) inability to read and speak Hungarian,3) outside of age range,4) prior placenta removal or acute cervical cancer treatment,5) survey form returned with missing data on key variables.

#### Ethics

The research was approved by the ethics committee of the Faculty of Health, University of Pécs before implementation (decision # ETKB/PTE-ETK/35–2019). Census data indicating Roma origin was obtained by permission from the Bureau of Statistics. Participant list with identification was deleted after making the mail contact. Participation and final data collection were anonym and voluntary. All participants received and signed an informed consent. No monetary or other incentives were used to solicit responses. Completed survey forms were mailed in by pre-paid envelopes. Nurses received no compensation for research and participant support.

#### Statistics

Descriptive statistics was used to describe sample characteristics. One-sample Kolmogorov–Smirnov test was employed to evaluate normal distribution of main measures. In case of non-normal distribution, Spearman rank correlation coefficients were calculated to establish associations between main variables. Bivariate logistic regression analysis was used to predict actual screening participation and HPV vaccination. All independent variables were entered in a single step to perform the analysis. Multicollinearity of the independent variables was checked by means of running multiple linear regression analysis and evaluating the variance inflation factors (VIF values). The range of values was between 1.20–1.43 indicating no influence of multicollinearity). Outlier detection (standard residuals >  ± 2.0) was performed and outliers outside the range of values were removed from final analysis (a total of 19 outliers were removed). A priori sample size calculations (based on a one-tailed test with significance = 0.5; power = 0.8 and odds ratio = 2.0) indicated a total of 110 subjects required [27]. Missing data were excluded from analysis, no data replacement was performed. Actual past attendance of cervical screening was used as the dependent variable. A total of seventeen items of our instrument, representing scale dimensions described above, were utilized as independent variables for the analysis.

## Results

Table 1 displays sample characteristics. The average age of our sample was 36.4 years (SD 11.3). A total of 9% lived alone while 81% lived with some significant other (husband, relatives or children). In terms of education, 61.4% had less than or equal to primary school degree, 33.4% held high school degree and 5.2% graduate or postgraduate. Of the total sample, only 62.5% had active employment. As for financial status, 36.1% said their financial situation was below average, 62% thought they had average income, and 1.9% reported above average standards of living. Finally, 17.4% of women attended at least one cervical screening and HPV vaccination in the past. When asked about the first time it happened, the average age was 21.1 (SD 6.9) years, 14 years of age being the youngest and 54 the oldest.

**Table 1:** Descriptive statistics

	N	Minimum	Maximum	Mean	Std. Deviation
Age	368,00	17,00	63,00	36,43	11,27
What age did you attend screening first?	299,00	14,00	54,00	21,14	6,97
Self-reported health	368,00	1,00	5,00	2,96	0,97
Full scale score	359,00	17,00	60,00	47,00	7,49
Family status	Frequency	Percent			
Married/co-habiting	213	57,9			
Single	88	23,9			
Divorced	44	12,0			
Widowed	23	6,3			
Total	368	100,0			
Who do you live with?	Frequency	Percent			
Live alone	33	9,0			
Co-habiting	40	10,9			
Co-habiting + children	128	34,8			
Co-habiting + children + other relatives	46	12,5			
Live with children	64	17,4			
Live with other relatives	57	15,5			
Total	368	100,0			
Education	Frequency	Percent			
Less than primary school	226	61,4			
Primary school	68	18,5			
Secondary/vocational school	55	14,9			
Gradute/university	19	5,2			
Total	368	100,0			
Employment	Frequency	Percent			
With job	230	62,5			
Without a job	138	37,5			
Total	368	100,0			
Financial status	Frequency	Percent			
Below average	133	36,1			
Average	228	62,0			
Above average	7	1,9			
Total	368	100,0			
Have you attended screening?	Frequency	Percent			
No	303	82,3			
Yes	64	17,4			
Total	367	99,7			
Missing	1	0,3			
Total	368	100,0			
Frequency of screening	Frequency	Percent			
Annual	199	52,5			
Every 2 years	52	14,1			
Every 3 years	23	6,3			
More than 3 years	61	16,6			
Total	329	89,4			
Missing	39	10,6			
Total	368	100,0			

Considering self-reported health of participants, average health rating (on a 5-point scale) was 2.96 (SD 0.96). The average score on the full screening attitude scale was 47.00 (SD 7.49).

Table 2 presents outcomes of correlation analyses. In terms of the association between self-reported health and employment, those actively employed enjoyed better health. Education was also positively associated with health; greater education was linked to better health. Income however did not correlate with past screening attendance. Self-reported health however did not relate to past screening. Education, however, was positively associated with past attendance, greater education increased screening attendance. The fact that our respondent did not co-habit with someone had no influence on past screening attendance. Future screening attendance was positively associated with income and education; more income and greater education increased the potential of attending future screening (r_income_ = 0.17, *p* < 0.001, r_education_ = 0.19, *p* < 0.001).

**Table 2:** Correlation analyses

Variable 1	Variable 2	correlation coeff. (r)	sig
self-reported health	employment	0.18	< 0.001
self-reported health	education	0.35	< 0.001
self-reported health	past screening attendance	0.14	0.39
education	past screening attendance	0.19	< 0.001
income	past screening attendance	0.84	0.53
co-habiting	past screening attendance	0.44	0.20
past negative experience	racially unfair treatment by physicians	0.48	< 0.001
past negative experience	community nurses performing screening	-0.27	< 0.001

Past negative screening experience was strongly correlated to doctors treating respondents racially unfair; the more unfair the personal conduct had been, the more past experience was appraised negatively. The opposite was true for community nurses; the more community nurses had been involved in screening, the less respondents reported negative experiences.

Finally, we preformed binary logistic regression predicting previous cervical screening attendance and HPV vaccination. The full model was significant (𝒳^2^ = 121.96, *p* < 0.001, -2LL = 156.96) and resulted in an 87.9% correct classification and a good model fit. The model explained 44% of the variance in HPV screening/vaccination attendance. Table 3 presents outcomes of the logistic regression model. Variables that significantly predicted past cervical screening attendance were friends having cervical cancer, community nurse performing the check-up and vaccination, respondent feeling no shame, procedure being painful, respondent only financially motivated and had a negative prior experience, and less travel involved. Variables that made the biggest contribution to past screening attendance, expressed by the absolute value of the odds (odds ratio) in the model, were past negative experience (4.503), community nurse involvement (3.325) and feeling shameful (1,609).

**Table 3:** Logistic regression model of screening/vaccination attendance (dependent variable: Have you ever attended cervical screening and received HPV vaccine? [yes/no])

**Variables that reached significance:**	Odds ratio	Sig
*“I attend cervical screening and HPV vaccination because…”*		
…of cervical cancer in my friends	0.472	**0.002**
…my community nurse will do sampling and vaccination	3.325	** < 0.001**
…I feel no shame	1.609	**0.043**
…I had no negative experience before	4.503	** < 0.001**
…I don't have to travel a lot	0.592	**0.012**
I don't attend screening/vaccination because it is too painful	0.509	**0.009**
I can only financially be motivated to attend cervical screening/vaccination	0.602	**0.037**
**Variables that did not reach significance:**	Odds ratio	Sig
*“I attend cervical screening and HPV vaccination because…”*		
…I care about my health	0.665	0.089
…of cervical cancer in my family	1.433	0.101
…I want to prevent cervical cancer	0.890	0.634
…my general practitioner told me to do so	0.826	0.349
…the doctor does not treat me racially unfair	1.309	0.202
…sampling and vaccination will be done by the physician in his (GP) office	0.987	0.944
…it is organized by my workplace	0.737	0.095
I don't attend screening/vaccination because it is too expensive	0.859	0.530
I have no time for screening/vaccination because of family and other obligations	0.829	0.371
I have no trust in cervical screening/vaccination	1.040	0.854
Constant	1 205.580	0.000

## Discussion

The main objective of the current research was to predict past cervical screening attendance and HPV vaccination in a sample of Roma women. Past cervical screening attendance among Roma women was 17.4% which is not significantly lower compared to the general Hungarian female population (22.0–23.3%) [28]. However, when we compared our 3-year screening coverage to the general population, numbers fell unfavorably for our sample (7% vs 52.6%) [28].

As expected, we supported that self-reported health of respondents was positively correlated with greater income and education. We found that income and health were not linked to past screening attendance or HPV vaccination. Greater education, which we attributed to increased knowledge about health, was positively linked to past screening and vaccination. When considering our screening attitude assessment, both income and education was positively linked to screening attendance and vaccination (more income and better education increased turnout probability). These results were in support of Adjei Boakye et al. [3], Jeudin et al. [11], Marlow [4] and Riza et al. [7].

Our logistic regression model predicted past screening and vaccination with increased precision (88% correct classification). The greatest odds of attending screening/vaccination was attributed to ‘no negative prior experience’. Those who did not have such experience were 4.5 times more likely to seek screening/vaccination than those who had a negative past influence. In order of magnitude, community nurse performing the process was the second most influential variable. Those who favored the community nurse performing the check-up/vaccination were 3.3 times more probable to participate. The third most influential variable was ‘feeling no shame’. Those who agreed to ‘feeling no shame’ about the procedure were 60% more prone to attend screening/vaccination compared those who felt ashamed. All three findings confirm and support Getrich et al. [14] and Vu et al. [20] who argued that the quality of the patient-provider relationship was a critical aspect of screening and immunization preference.

Appraising the screening/vaccination process painful, receiving financial compensation to attend, and attendance involving a lot of travel decreased the odds of ‘no attendance’ by 50%, 40% and 41%, respectively. When respondents’ friends suffered of cervical cancer, the odds of screening attendance/vaccination dropped by 53%. The result is opposite to expectations and authors have no immediate explanations for this outcome. More in depth research is suggested to explore the background of this behavior.

Note that while community nurse involvement was a significant determinant of HPV screening/vaccination, ‘doctors acting racially unfair’ as well as ‘sampling performed by the physician in his (GP) office’ did not make any contribution to explaining attendance. The same was true for ‘preventive reasons’ and ‘cancer in the family’, which did not reach significance in our model. These outcomes may have to do with cultural norms, but further research is recommended to confirm the assumption.

## Conclusion

Referring to the ratio of past cervical screening attendance, we conclude that our female Roma sample did not behave differently from the general population. We saw evidence that racial mistreatment negatively influenced cervical screening/vaccination participation, but when weighed together with other variables, racial mistreatment did not emerge as a significant predictor of screening attendance. Not feeling shameful about the screening procedure increased the odds of screening/vaccination participation. The positive role of the community nurse and her involvement in the procedure was strongly emphasized by respondents. Having no negative prior experience with screening increased the odds of participation the most. Cervical cancer of friends, procedural pain, financial motivation and travel distance all decreased odds of participation but to a much lesser extent.

In order to improve future screening and immunization, community nurses should play more central and advanced role in the organization and implementation of such services that specifically target Roma populations.

### Limitations

Only Roma women with permanent address were involved. While this may have introduced a sampling bias, it is estimated that less than 2% of the total Roma population living in Hungary has no registered address [29]. While only individual scale items were used for statistical analysis, and while we demonstrated sufficient reliability for the self-developed screening attitude scale, authors acknowledge that the instrument was not validated for this research. Regression results may have been different had we used 3-year screening attendance as the dependent variable, however, sample size would have been insufficient to provide enough statistical power to make valid inferences. Missing data was only a minor issue since data were obtained from 368 women in total and complete data from 359 subjects were used for statistical analyses. Authors also admit that generalizability of results may be limited to Central Eastern European Roma populations as both health systems and Roma cultures differ between the Eastern and Western parts of Europe.

## Data Availability

The datasets used and/or analyzed during the current study are available from the corresponding author on reasonable request.

## Abbreviations

GP: General Practitioner
HPV: Human papillomavirus
VIF: Variance inflation factor
